# Longitudinal stent elongation or shortening after deployment in the coronary arteries: which is dominant?

**DOI:** 10.1186/s43044-021-00170-9

**Published:** 2021-05-17

**Authors:** Magdy Algowhary, Mohammed Aboel-Kassem F. Abdelmegid

**Affiliations:** Department of Cardiovascular Medicine, Assiut University Heart Hospital, Faculty of Medicine, Assiut University, Asyut, 71515 Egypt

**Keywords:** IVUS, Stent length, Stent elongation and shortening

## Abstract

**Background:**

Stent manufacturers always record stent shortening data while they do not record stent elongation data. The aim of this study is to identify both stent shortening and elongation occurring after deployment in the coronary arteries and know their percentage.

**Results:**

The length of coronary stents was measured by intravascular ultrasound (IVUS) by (1) edge-to-edge (E-E) length, measured from the appearance of the first distal strut to the last proximal strut, and (2) area-to-area (A-A) length, measured from the first distal struts seen at more than one IVUS quadrant to the last proximal struts seen at more than one IVUS quadrant. Stent shortening was defined as both E-E and A-A lengths were shorter than the manufacturer box-stated length (shortened group). Stent elongation was defined as both E-E and A-A lengths were longer than the manufacturer box-stated length (elongated group), otherwise unchanged group. Consecutive 102 stents deployed in ischemic patients were included. Stent elongation was detected in 67.6% (69 stents), and shortening was detected in 15.7% (16 stents), while unchanged stents were detected in 16.7% (17 stents). Although the 3 groups had similar box-stated length and predicted foreshortened length, they had significantly different measurements by IVUS, *p<*0.001 for each comparison. Differences from box-stated length were 1.9±1.4mm, −1.4±0.4mm, and 0.4±0.3mm, respectively, *p<*0.001. The elongated group had significantly longer differences from the corresponding box-stated and predicted foreshortened lengths, while the shortened group had significantly shorter differences from the corresponding box-stated length and similar foreshortened length. By multinomial regression analysis, the plaque-media area and stent deployment pressure were the independent predictors of the stent length groups, *p=*0.015 and *p=*0.026, respectively.

**Conclusions:**

Change in stent length is not only shortening—as mentioned in the manufacturer documents—but also stent elongation. Stent elongation is dominant, and the most important predictors of longitudinal stent changes are plaque-media area and stent deployment pressure.

## Background

Coronary stenting is an important tool in the management of coronary artery disease. Understanding stent geometry is essential to treat different coronary lesions. The researchers studied stent diameter and found that the minimal stent diameter measured by intracoronary ultrasound (IVUS) was significantly smaller than that predicted in the manufacturers’ compliance charts, and these differences were independent of stent manufacturers, deployment pressure, or stent length [1, 2] but may be related to vessel calcium [3]. The longitudinal stent dimension is a subject for further studies. Stent foreshortening defined as the difference between the desired and the actual stent length after deployment is a part of these studies. Cases with radiologic overt stent compression and elongation after deployment are reported and termed longitudinal stent deformation (LSD) [4]. A variety of mechanisms are responsible for LSD. All of them are mechanical in origin such as the impact of guiding catheter tip or following passage of catheters, guidewire, post-dilatation balloon, embolic protection devices, and IVUS catheters [5]. In the past, it was reported after deployment of coil stents such as Wiktor stent [6, 7] and more recently after deployment of Promus, Xience, and Endeavor stents [8–14]. LSD affects coronary blood flow leading to stent thrombosis [5], target lesion failure [14], and even death [7]. Of note, it is not evident in the majority of cases [5, 9]; however, stent shortening and elongation are frequently seen but at a less magnitude. Stent shortening is evident in self-expandable stents so that the stent with enough length should be used to cover the whole lesion site. It is also evident in balloon-mounted stents [15]. On the other side, stent elongation is recorded. Although in recent trials using IVUS examination stent elongation could be seen after deployment of balloon-mounted stents [16], still stent foreshortening is the only longitudinal stent change mentioned in stent manufacturer documents.

The aim of this study is to confirm the presence of stent elongation and/or shortening and its percentage in reality after deployment of balloon-mounted stents in coronary lesions providing that there is no LSD problem detected by both angiographic and IVUS examinations.

## Methods

### Study population

Consecutive patients with stable, unstable angina pectoris and myocardial infarction due to de novo coronary artery disease were included in this study. All patients who underwent a successful IVUS-guided percutaneous coronary intervention were included in this study. The study was conducted on real-world patients after a written informed consent was taken from every patient who underwent cardiac catheterization. All procedures performed in studies involving human participants were in accordance with the ethical standards of the Helsinki Declaration. The lesions were selected based on angiographic examination. To avoid incorrect stent length measurements, lesions were included if they had no acute angulation, no tortuosity, no ectasia, and no heavy calcification. Moreover, lesion type C, bifurcation lesion, small vessel disease (reference vessel size less than 2.0mm), and ostial lesion were excluded from the analysis. Lesions located at the side branch should be totally covered by the stent away from its ostium. Both drug-eluting stents (DES) and bare-metal stents (BMS) were included according to the operator’s decision. Six types of stents were used: Multilink (Abbott, IL, USA), Integrity (Medtronic, MN, USA), Commander (Alvimedica, IS, Turkey), Promus (everolimus-eluting stent, Boston, MA, USA), Resolute (Zotarolimus eluting stent, Medtronic, MN, USA), and Cre8 (sirolimus-eluting stent, CID, VC, Italy). Small stents, <3.0mm in diameter, were not included in the analysis. Patients with a two-vessel disease were also included as long as one stent per lesion was used. Patients were excluded when the lesions were treated by two overlapped stents, in-stent restenosis, poor IVUS image quality, manual pullback, non-uniform or interrupted IVUS pullback, and cases with radiologic longitudinal stent deformation or fracture. All patients gave informed consent and that the authors have conformed to the institutional guidelines.

On the basis of diagnostic coronary angiography, patients underwent percutaneous coronary intervention if one or more major coronary arteries had a stenosis of at least 70% and were suitable for revascularization. Anti-platelets and heparin were administered before the procedures. Balloon-mounted stents were deployed directly or after balloon dilatation. No exclusion was made for stent type, strut thickness, stent recoil, stent metal/artery ratio, or the number of strut connectors. Coronary stenting was guided by IVUS examination before and after stenting. If incomplete stent expansion/malapposition was detected, further balloon dilatation was done to achieve optimal stenting results.

### IVUS procedure and analysis

Examination was performed using a 2.5-F IVUS catheter operating on a frequency of 40 MHz after administration of intracoronary nitrates, 200mcg. The transducer was positioned in the distal vessel, at least 10mm distal to the stent, and withdrawn at a rate of 0.5–1.0 mm/s with the use of a motor drive (CardioVascular Imaging System ClearView Ultra, CVIS, Boston Scientific, Fremont, CA, USA) to the aorto-ostial junction. On a computer screen, manual planimetry was performed to measure the external elastic membrane (EEM = vessel area (VA)) and lumen areas (LA) in all frames. Since the IVUS catheter may not pass through the center of the stent in all examinations, the transverse plane of stent edges may not identically the same as the transverse plane of the vessel. Cases with study images that had an oval-shaped image or distorted image were not included. Moreover, to minimize the effect of this disposition on the accuracy of stent length measurements, stent length was derived by 3 methods: (1) edge-to-edge (E-E) stent length which was the distance measured at the long axis from the first distal frame with the first stent strut located at one quadrant seen at the short axis to the proximal frame with the last stent strut located at one quadrant, (2) area-to-area (A-A) stent length which was the distance measured at the long axis from the first distal frame with the first stent struts located at two or more quadrants seen at the short axis to the proximal frame with the last stent struts located at two or more quadrants, and (3) average stent length: [(E-E) length + (A-A) length]/2. The distance located between the start of E-E length to the A-A length on both ends was not an exclusion criterion. Details of the definitions and measurements of E-E, A-A, and average lengths used as parameters for the stent length were explained before by the work of Dvir et al. published in 2014 [16]. Longitudinal stent length was considered as follows: (1) elongated if both E-E length and A-A length were longer than manufacturer’s box-stated length, (2) shortened if both E-E length and A-A length were shorter than the manufacturer’s box-stated length, and (3) unchanged if it was neither 1 nor 2.

### Statistical analysis

Categorical variables are presented as frequencies. Continuous variables are reported as mean ± SD for normally distributed variables and median [interquartile range (IQR)] for variables without a normal distribution pattern. The chi-square with Fisher’s exact tests were used for comparisons of categorical variables. For normally distributed variables, the *T* tests (paired and independent samples) were used to measure equality of the means between 2 groups. One-way ANOVA was used for group comparison, and the Bonferroni and Tamhane methods were used for post hoc comparisons. For nonparametric comparisons, the Wilcoxon, Mann-Whitney, and Kruskal-Wallis tests were used. For correlations, the Pearson and Spearman tests were used. To identify the predictors of the stent length groups (shortened, unchanged, and elongated stents), the multinomial logistic regression analysis with stepwise forward entry method was used. The following variables were entered in the model: smoking, ejection fraction, deployment pressure groups, DES/BMS, deployed stents, stent diameter, manufacturer predicted foreshortened stent length, lesion length, minimal lumen diameter, lesion plaque type by IVUS, distal edge plaque type by IVUS, reference LA by IVUS, lesion VA, lesion LA, lesion plaque-media area, proximal edge VA, proximal edge LA, proximal edge plaque-media area, distal edge VA, and distal edge plaque-media area. All tests were performed by using the SPSS package version 25 (SPSS Inc., Chicago, IL, USA). The statistical tests were two-sided, and *p<*0.05 was considered statistically significant.

## Results

Consecutive 102 balloon-mounted coronary stents were used to treat ischemic patients; their age ranged from 40 to 84 years old; 84.3% were males (86 patients), 64.7% were hyperlipidemic (66 patients), 62.7% were hypertensive (64 patients), 52.9% were smokers (54 patients), and 33.3% were diabetic (34 patients). Tables 1 and 2 show the patients’ clinical, angiographic, and IVUS data by stent groups. The elongated stents represent 67.6% (69 stents), the shortened stents represent 15.7% (16 stents), and the unchanged stents (similar to manufacturer stent box-stated length) represent 16.7% (17 stents). Apart from smoking, stent type (DES and BMS), and stent diameter, the 3 stent groups have no statistically significant differences regarding clinical, angiographic, and stenting data. Deployed stents, stent length (from 8 to 38mm), stent metal/artery ratio, stent recoil percentage, strut thickness, manufacturer stent box-stated length, and stent foreshortening are not significantly different while IVUS data shows mixed parameters (Tables 1 and 3). Reference VA, reference LA, proximal LA, distal LA, eccentric lesion plaque, and plaque characters at both stent edges are not significantly different. Lesion plaque-media area, proximal plaque-media area, and plaque characters at the lesion site tend to be significantly different. The 3 groups have significantly different measurements regarding proximal VA, lesion VA, lesion LA, distal VA, and distal plaque-media area. By post hoc analysis, the elongated stent group has a significantly smaller vessel area than the unchanged stent group at the lesion site, *p*=0.003; proximal site, *p*=0.01; and distal site, *p<*0.001. Also, it has a significantly smaller lumen area at the lesion site, *p*=0.01, and a smaller plaque-media area at the distal site, *p*<0.001.

**Table 1:** Baseline characteristics by groups

		Elongated stents	Shortened stents	Unchanged stents	***p value***
		***(n*** = 69, 67.6%)	(***n*** = 16, 15.7%)	(***n*** = 17, 16.7%)	
Age, years		65.6 ± 10.0	67.6 ± 10	64.8 ± 11.6	0.72
Males		57 (82.6%)	14 (87.5%)	15 (88.2%)	0.92
Smoking		30 (43.5%)	12 (75%)	12 (70.6%)	0.043
Hypertension		42 (60.9%)	12 (75%)	10 (58.8%)	0.77
DM		22 (31.9%)	8 (50%)	4 (23.5%)	0.37
Dyslipidemia		45 (65.2%)	11 (68.8%)	10 (58.8%)	0.8
Family history		7 (10.1%)	4 (25%)	3 (17.6%)	0.28
Clinical presentation					0.27
	AP	29 (42%)	7 (43.8%)	6 (35.3%)	
	UAP	20 (29%)	7 (43.8%)	3 (17.6%)	
	AMI	20 (29%)	2 (12.5%)	8 (47.1%)	
Statin		41 (59.4%)	9 (56.3%)	7 (41.2%)	0.27
Beta blockers		17 (24.6%)	7 (43.8%)	5 (29.4%)	0.39
Calcium antagonists		26 (37.7%)	9 (56.3%)	7 (41.2%)	0.65
ACE-I		16 (23.2%)	5 (31.3%)	4 (23.5%)	0.94
ARB		20 (29%)	4 (25%)	8 (47.1%)	0.28
EF		62.9 ± 11.6	64.0 ± 13.7	55.0 ± 14.7	0.061
Cholesterol, mg/dL		189.5 ± 32.6	192.7 ± 39.8	189.4 ± 47.3	0.95
HDL-cholesterol, mg/dL		49.4 ± 12.6	47.4 ± 12.6	45.3 ± 18.3	0.61
LDL-cholesterol, mg/dL		121.9 ± 31.1	116.2 ± 13.4	114.8 ± 26.3	0.85
Angiographic data:					
Stented vessel					0.37
	LAD	40 (58%)	11 (68.8%)	8 (47.1%)	
	LCX	13 (18.8%)	4 (25%)	3 (17.6%)	
	RCA	16 (23.2%)	1 (6.3%)	6 (35.3%)	
ACC/AHA lesion type					0.45
	A	31 (44.9%)	8 (50%)	9 (52.9%)	
	B	31 (44.9%)	8 (50%)	8 (47.1%)	
	C	7 (10.1%)	0	0	
Lesion length, mm		11.3 ± 6.4	9.4 ± 3.8	8.8 ± 3.2	0.18
Reference diameter, mm		3.1 ± 0.6	3.1 ± 0.7	3.3 ± 0.7	0.45
MLD, mm		0.8 ± 0.3	0.8 ± 0.3	1.0 ± 0.2	0.15
DS%		74.1 ± 16.3	71.2 ± 14.9	70.9 ± 6.9	0.65
Deployment pressure, atm		16.7 ± 3.2	15.1 ± 3.4	16.1 ± 2.8	0.19
High pressure stenting, >16 atm		49 (71%)	7 (43.8%)	10 (58.8%)	0.105
Post-stent baloon dilatation		20 (29%)	2 (12.5%)	4 (23.5%)	0.49
IVUS data:					
Reference VA, mm2		15.4 ± 3.8	14.9 ± 3.3	17.4 ± 4.6	0.26
Reference LA, mm2		9.3 ± 2.2	8.9 ± 2.6	10.8 ± 2.8	0.14
Proximal site VA, mm2		16.6 ± 5.0*	17.0 ± 4.3	21.7 ± 4.8	0.02
Proximal site LA, mm2		8.7 ± 3.2	9.0 ± 2.6	11.0 ± 4.3	0.15
Proximal site PMA, mm2		7.9 ± 3.2	8.0 ± 2.8	10.7 ± 4.3	0.064
Lesion VA, mm2		13.2 ± 4.0*	14.3 ± 4.0	17.6 ± 5.0	0.004
Lesion LA, mm2		4.0 ± 1.9*	4.4 ± 1.9	5.7 ± 2.0	0.016
Lesion PMA, mm2		9.2 ± 3.4	9.9 ± 3.3	11.9 ± 5.2	0.063
Distal site VA, mm2		10.8 ± 3.4*	12.0 ± 3.9	15.6 ± 3.2	<0.001
Distal site LA, mm2		6.1 ± 2.1	6.5 ± 2.0	6.9 ± 2.2	0.45
Distal site PMA, mm2		4.7 ± 2.4*	5.5 ± 2.8	8.8 ± 2.9	<0.001

Data provided as mean ± SD or number (%)

*ACC/AHA* American College of Cardiology and American Heart Association, *ACE-I* angiotensin converting enzyme inhibitors, *AMI* acute myocardial infacrtion, *AP* stable angina pectoris, *ARB* angiotensin receptor blockers, *CRP* C-reactive protein, *DES* drug-eluting stent, *DM* diabetes mellitus, *DS%* diameter stenosis percentage, *EF* ejection fraction, *HDL-C* high density lipoprotein cholesterol, *IVUS* intravascular ultrasound, *LA* lumen area, *LAD* left anterior descending artery, *LCX* left circumflex artery, *LDL-C* low density lipoprotein cholesterol, *MLD* minimal lumen diameter, *PMA* plaque-media area, *RCA* right coronary artery, *UAP* unstable angina pectoris, *VA* vessel area

*p<0.05, compared to unchanged stents

**Table 2:** Stent types and characters by groups

	Elongated stents (*n* = 69, 67.6%)	Shortened stents (*n* = 16, 15.7%)	Unchanged stents (*n* = 17, 16.7%)	*p* value
Stent type, %				0.01
DES	38 (55.1%)	4 (25%)	3 (17.6%)	
BMS	31 (44.9%)	12 (75%)	14 (82.4%)	
Stent box-stated length, mm				0.34
Less than 20mm	45 (65.2%)	10 (62.5%)	14 (82.4%)	
More than 20mm	24 (34.8%)	6 (37.5%)	3 (17.6%)	
Stent diameter, mm	3.0 (3.0–3.0)	3.0 (3.0–4.0)	4.0 (3.0–4.0)*	0.01
Stent strut thickness, micro	81.0 (81.0–91.0)	81.0 (81.0–88.5)	81.0 (81.0–86.0)	0.69
Stent metal/artery ratio	19.0 (14.1–19.9)	19.0 (19.0–19.9)	19.0 (19.0–19.5)	0.81
Stent recoil (%)	4.0 (3.0–4.9)	4.0 (3.0–4.0)	4.0 (3.5–4.0)	0.94

Data provided as number (%) or median (IQR)

*BMS* bare-metal stents, *DES* drug-eluting stents

*p=0.002, compared to the elongated group

**Table 3:** IVUS plaque-media type by groups

	Elongated stents (*n* = 69, 67.6%)	Shortened stents (*n* = 16, 15.7%)	Unchanged stents (*n* = 17, 16.7%)	*p* value
At proximal edge				0.39
Soft	14 (20.3%)	3 (18.8%)	5 (29.4%)	
Mixed	21 (30.4%)	5 (31.3%)	5 (29.4%)	
Hard	4 (5.8%)	1 (6.3%)	1 (5.9%)	
Superficial calcification	19 (27.5%)	3 (18.8%)	3 (17.6%)	
Deep calcification	11 (15.9%)	4 (25%)	3 (17.6%)	
At lesion site				0.06
Soft	13 (18.8%)	1 (6.3%)	2 (11.8%)	
Mixed	15 (21.7%)	5 (31.3%)	5 (29.4%)	
Hard	6 (8.7%)	5 (31.3%)	2 (11.8%)	
Superficial calcification	15 (21.7%)	3 (18.8%)	2 (11.8%)	
Deep calcification	20 (29%)	2 (12.5%)	6 (35.3%)	
Eccentric lesion plaque				0.98
Yes	32 (46.4%)	9 (56.3%)	6 (35.3%)	
At distal edge				0.11
Soft	17 (24.6%)	3 (18.8%)	2 (11.8%)	
Mixed	10 (14.5%)	5 (31.3%)	5 (29.4%)	
Hard	5 (7.2%)	2 (12.5%)	1 (5.9%)	
Superficial calcification	22 (31.9%)	3 (18.8%)	5 (29.4%)	
Deep calcification	15 (21.7%)	3 (18.8%)	4 (23.5%)	

Data provided as number (%)

*IVUS* intravascular ultrasound

**Fig. 1:**
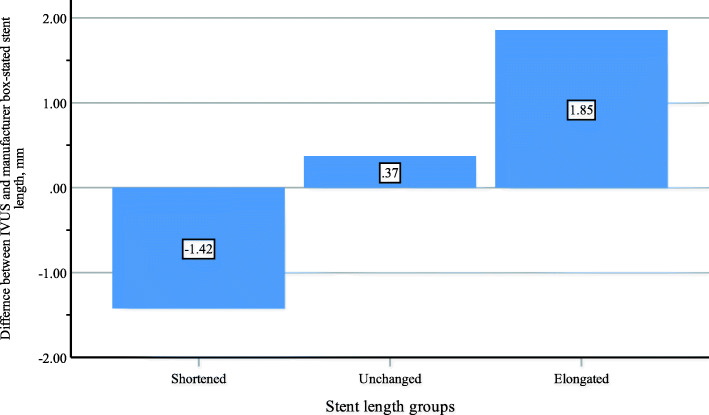
Groups of stent length classified by intravascular ultrasound

The stent box-stated length is highly correlated with IVUS measurements of stent lengths, for all correlations *r*=0.96 and *p<*0.001. Manufacturer stent length data including box-stated length and predicted foreshortened length are comparable among the 3 groups (Table 4) while IVUS stent length measurements regarding E-E and A-A lengths tend to be different, and the average length is significantly different, *p=*0.01. The median average stent length is significantly longer in the elongated group than in both the shortened and the unchanged groups, *p=*0.03 and *p=*0.01, respectively. Moreover, the differences in length between IVUS measurements (E-E, A-A, and average lengths) and manufacturer lengths (box-stated length and predicted foreshortened lengths) are significant among the 3 groups, *p<*0.001 for each comparison (Fig. 1). The difference between E-E length and box-stated length is significantly longer in the elongated group than in both the shortened and unchanged groups, *p<*0.001 and *p=*0.004, respectively. Also, it is significantly shorter in the shortened group than in the unchanged group, *p=*0.008. Similarly, the difference between A-A length and the box-stated length is significantly longer in the elongated group than in both the shortened and unchanged groups, *p<*0.001 for each comparison; consequently, the median difference between the average IVUS length and the box-stated length is significantly longer in the elongated group than in both the shortened and unchanged groups, *p<*0.001 for each comparison, and significantly shorter in the shortened group than unchanged group, *p=*0.001. The difference between E-E length and predicted foreshortened stent length in the elongated group is significantly longer than the difference in the shortened and in unchanged groups, *p<*0.001 and *p=*0.02, respectively. Similarly, the difference between A-A length and predicted foreshortened stent length is significantly longer in the elongated group than the others, *p<*0.001 and *p=*0.004, respectively; consequently, the median difference between the average length and the predicted foreshortened stent length is significantly longer in the elongated group than in the shortened and unchanged groups, *p<*0.001 for each comparison, and it is significantly shorter in the shortened group than in the unchanged group, *p=*0.001.

**Table 4:** IVUS and manufacturer stent lengths by groups

	Elongated stents (*n* = 69, 67.6%)	Shortened stents (*n* = 16, 15.7%)	Unchanged stents (*n* = 17, 16.7%)	*p* value
Manufacturer stent length measurements				
Box-stated stent length, mm	19.3 ± 5.7	19.9 ± 4.7	17.7 ± 6.4	0.49
Predicted forshortened stent length, mm	18.4 ± 4.9	19.5 ± 4.7	16.0 ± 3.8	0.1
IVUS stent length measurements				
(E-E) stent length, mm	21.2 ± 6.2	18.5 ± 4.3	18.1 ± 6.5	0.08
(A-A) stent length, mm	20.7 ± 6.3	18.2 ± 4.4	17.2 ± 6.6	0.06
Average IVUS stent length, mm	19.7 (16.2–24.3)*	17.4 (14.7–22.7)	17.7 (14.7–18.3)	0.01
Stent length differences (IVUS-manufacturer)				
(E-E) stent length - box-stated stent length, mm	1.9 ± 1.4*	(−)1.4 ± 0.4^†^	0.4 ± 0.3	<0.001
(A-A) stent length - box-stated stent length, mm	1.4 ± 1.3*	(−)1.8 ± 0.3	(−)0.5 ± 0.4	<0.001
Average IVUS stent length - box-stated stent length, mm	1.3 (0.7–2.2)*	(−)0.5 [(−)1.1–(−)0.4]^†^	(−)0.1 [(−)0.3–0.2]	<0.001
(E-E) stent length - predicted foreshortened stent length, mm	1.84 (1.3–2.8)*	0.65 (0.5–1.1)	(−)0.2 [(−)0.5–0.3]	<0.001
(A-A) length - predicted foreshortened stent length, mm	1.3 (0.8–2.3)*	(−)0.04 [(−)0.4–0.2]	(−)0.4 [(−)0.8–(−)0.1]	<0.001
Average IVUS stent length - predicted foreshortened stent length, mm	1.6 (1.0–2.6)*	(−)0.3 [(−)0.7–0.6]^†^	0.3 (0.1–0.5)	<0.001

Data provided as mean ± SD or median (IQR)

*A-A* area-to-area length, *E-E* edge-to-edge length, *IVUS* intravascular ultrasound

*p<0.05, compared to both shortened and unchanged stents

^†^*p<*0.05, compared to unchanged stents

Table 5 shows the measurements of the stent length by IVUS compared to corresponding manufacturer box-stated stent length. In the elongated stent group, the E-E, A-A, and average lengths are significantly longer than their corresponding box-stated stent length, *p<*0.001 for each comparison. Also, in the shortened stent group, they are significantly shorter than their corresponding box-stated measurements, *p=*0.05, *p=*0.04, and *p=*0.001, respectively.

**Table 5:** IVUS stent lengths vs manufacturer box-stated stent length

	Box-stated length	E-E length	*p*	A-A length	p*	Average IVUS length	*p*^†^
Elongated stent group, mm	19.3 ± 5.7	21.2 ± 6.2	<0.001	20.7 ± 6.3	<0.001	19.7 (16.2–24.3)	<0.001
Shortened stent group, mm	19.9 ± 4.7	18.5 ± 4.3	0.05	18.2 ± 4.4	0.04	17.4 (14.7–22.7)	0.001

Data provided as mean ± SD or median (IQR)

*A-A length* area-to-area stent length, *E-E length* edge-to-edge stent length, *IVUS* intravascular ultrasound

*p denotes A-A stent length vs box-stated stent length

^†^*p* denotes average IVUS stent length vs box-stated stent length [in the elongated stent group = 19.7 (16.2–24.3) vs 18.0 (15–23) and in the shortened stent group = 17.4 (14.7–22.7) vs 18.0 (15.8–23.8)]

Table 6 shows the measurements of stent length by IVUS compared to the manufacturer-predicted foreshortened stent length. In the elongated stent group, the E-E, A-A, and average lengths are significantly longer than their corresponding predicted foreshortened stent length, *p<*0.001 for each comparison, while in shortened stent group, the E-E, A-A, and average lengths are comparable with their corresponding predicted foreshortened stent length.

**Table 6:** IVUS stent lengths vs predicted foreshortened stent length

	Predicted foreshortened length	E-E length	*p*	A-A length	p*	Average IVUS length	*p*^†^
Elongated stent group, mm	18.4 ± 4.9	21.2 ± 6.2	<0.001	20.7 ± 6.3	<0.001	19.7 (16.2–24.3)	<0.001
Shortened stent group, mm	19.5 ± 4.7	18.5 ± 4.3	0.19	18.2 ± 4.4	0.1	17.4 (14.7–22.7)	0.077

Data provided as mean ± SD or median (IQR)

*A-A length* area-to-area stent length, *E-E length* edge-to-edge stent length, *IVUS* intravascular ultrasound

*p denotes A-A stent length vs predicted foreshortened stent length

^†^*p* denotes average IVUS stent length vs predicted foreshortened stent length [in the elongated stent group = 19.7 (16.2–24.3) vs 17.8 (14.6–22.8) and in the shortened stent group = 17.4 (14.7–22.7) vs 17.6 (15.3–23.7)]

**Fig. 2:**
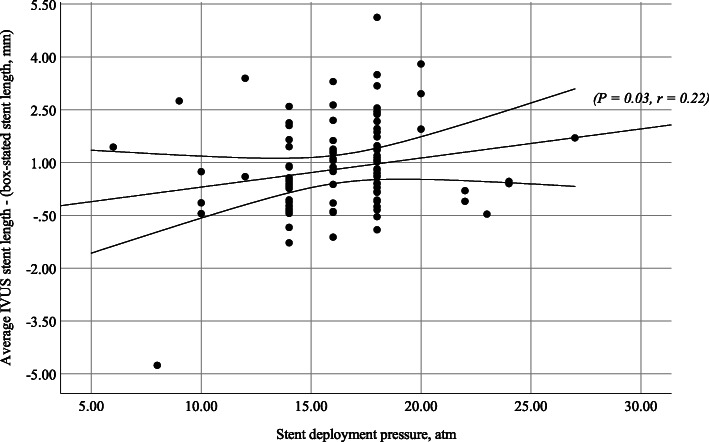
Correlation between stent deployment pressure and difference of stent length. A significant direct relation between stent deployment pressure and difference in stent length

**Fig. 3:**
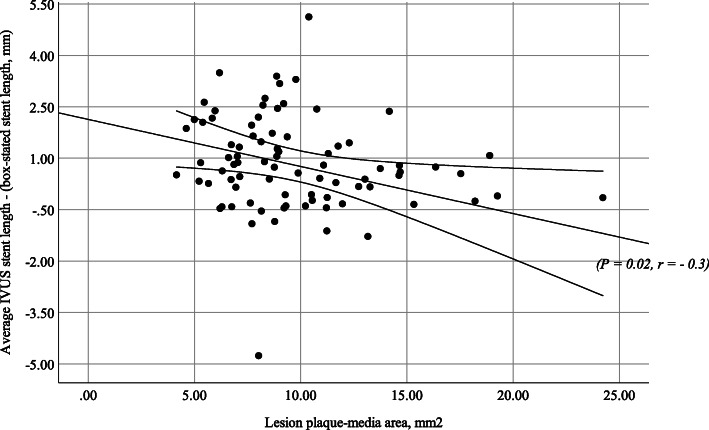
Correlation between the plaque-media area and difference of stent length. A significant inverse relation between plaque-media area and difference in stent length

The multivariate analysis model selects the stent deployment pressure groups, *p=*0.026, and plaque-media area, *p=*0.015, as the most significant independent predictors of stent length groups with a significant final fitting model (−2 log-likelihood ratio 49.28, *p=*0.003) and 56.3% correct classification. The difference of stent length between IVUS measurement and manufacturer length is correlated directly with deployment pressure and inversely with plaque-media area (Figs. 2 and 3).

## Discussion

The chief findings of this study were the presence of 3 forms of stent length after deployment in coronary artery lesions: elongated stents, shortened stents, and unchanged stents. Compared with the manufacturer box-stated length, 67.6% of the stents showed stent elongation without LSD confirmed by angiography and IVUS examination [10]. The rest of the stents were either unchanged stents (16.7%) which had similar manufacturer stent length data or shortened stents (15.7%) which had only similar manufacturer foreshortened stent length data. Stent elongation was the dominant finding in contrary to manufacturer data which stressed only on 2 forms: nominal and foreshortened stent lengths. This elongation was associated with stent deployment pressure and the amount of lesion plaque-media area.

The real-life change in stent length varies from −1.4mm (shortening) to 1.9mm (elongation). Stent elongation was seen in 67.7% of the stents (69 stents) while shortening was seen in 15.7% of the stents (16 stents); therefore, occurence of stent elongation was 4.3 times as much as stent shortening. Not only the incidence but also the quantity was greatly different. The median stent elongation exceeded 1.0mm [difference of average IVUS stent length and box-stated stent length = 1.3mm (0.7–2.2)], while the median shortening was very minimal {−0.5mm [(−1.1)–(−0.4)]}. If the overall median difference was not great, we noticed that 19.6% of studied stents, 20 stents, exceeded 2mm and reached 5.0mm in some stents, while shortening of more than 1mm was not seen except in 3.92% of all stents, 4 stents. Of course, this elongation was of concern though it was not mentioned by the stent manufacturers.

Nevertheless, elongated stents would have either beneficial or drawback effects. The possible beneficial effect would come from a complete covering of the lesion site giving good long-term results regarding restenosis. Also, the stent edges would be deployed in normal or less diseased proximal and distal reference segments decreasing the chance of stent edge restenosis. On the opposite side, the possible drawback would result from stent malapposition at the stent edge especially at the proximal reference segment which would be larger than the distal segment. If optimization would not be performed, stent thrombosis might occur, and consequently, myocardial infarction might happen. Moreover, an elongated stent may protrude into the nearby ostium of a side branch. On trying to deploy a stent in that nearby side branch, the protruded stent might obstruct the passage of the second stent to the side branch lesion. It might cause stent dislodgment or even embolization which would carry serious complications. This would be important in doing an intervention for a bifurcation lesion especially bifurcation of the left main coronary artery.

Although elongation was not related to the manufacture stent data (type, strut thickness, metal/artery, radial force), it was related to stent deployment pressure and plaque-media area. Of note, 92.1% of the study stents were deployed at a pressure more than 12 atm explaining why 67.6% of the stents were elongated. Recently, the expert consensus document of the European Association of Percutaneous Cardiovascular Intervention implies the importance of choosing stent length to cover the whole coronary lesion in order to avoid stent failure, stent thrombosis and restenosis, and occurrence of major adverse cardiac events such as myocardial infarction [17]. It also encourages the avoidance of landing stent edge within an area with plaque burden >50% to decrease the incidence of stent edge restenosis. In order to achieve full lesion coverage, we have to select ideal stent length by angiographic and other imaging modalities such as IVUS and optical coherence tomography (OCT). Knowing the manufacturer’s nominal stent length is mandatory for stent length selection keeping in mind that manufacturer foreshortening data is usually minimal. It is very important to choose stent length to avoid incomplete lesion coverage with its sequels because incomplete lesion coverage reaches up to 90% of lesions as documented by near-infrared spectroscopy and IVUS (NIRS-IVUS) [18]. It may be due to inaccurate stent length selection depending on angiographically measured lesion length alone and the occurrence of stent shortening. For this reason, manufacturer data always mention nominal and foreshortening data for each stent. Nevertheless, stent elongation data is usually not mentioned though recent studies of stent length have proved its existence [16, 18–22].

Not only in vivo human studies but also in vitro studies using bench models have shown stent elongation phenomenon in the current stents. It occurs under the influence of post-dilatation [4, 20, 23, 24]. Interestingly, linear stent elongation could be seen during each step of balloon dilatation depending on balloon dilatation pressure [23, 24] which is similar to our finding and the direction of dilatation [20]. More stent elongation could be detected when dilatation was performed in a distal to proximal direction [20]. Regarding in vivo studies, stent elongation has been documented in both first-generation DES (Cypher and Taxus stents) and second-generation DES (Xience V, Promus Element, and Endeavor stents) [16]. Similar to our results, the use of high deployment pressure is correlated with stent length difference. Recently, measuring the stent length by OCT after deployment in coronaries has revealed stent length elongation in series of DES (Xience series, Promus series, Ultimaster, Synergy, Integrity, and Coroflex stents) [19]. Post-balloon dilatation has been significantly associated with stent length differences too. Moreover, malapposition of the stent edge has been responsible for stent elongation after post-dilatation. The use of the proximal optimization technique (post-dilatation by a suitable balloon at the proximal edge rather than from the distal to proximal dilatation) has prevented stent elongation. This technique may be useful as it may prevent stent protrusion into the left main trunk during the intervention at the proximal portion of the left anterior descending branch or left circumflex branch [21]. Masuda et al. have explained that the use of post-dilatation balloon has resulted in generating longitudinal forces moving the stent longitudinally. However, proximal balloon dilatation may stop stent elongation as malapposition is usually encountered at the proximal edge [19] confirming the same in vitro results obtained recently by Sumi et al. [20]. The proximal optimization technique usually results in good stent apposition to the vessel wall preventing the stent from elongation in contrary to free stent edge with malapposition that will elongate easily. In the present study, multivariate analyses denote that deploying stent under high pressure is associated with stent elongation as it generates longitudinal forces pushing stent struts. Moreover, the presence of less plaque-media area at the lesion site and at the stent edges will allow easier longitudinal elongation because of less resistance encountered by the vessel wall. Lesion and edge plaque types regarding lipid-rich plaque, fibrous plaque, and calcific plaque are not a predictor of a difference in stent length in our study. Moreover, the site of calcification either superficial or deep is not a predictor too. The only important IVUS predictor is the plaque-media area at both lesion and edge locations. The explanation of the relation between plaque and stent length is not a simple relation or effect as has been illustrated in a model study performed by Wei et al. [25]. They demonstrated how complex the interaction between stent-plaque-artery. In his model, stent length was affected by plaque eccentricity and plaque compositions regarding lipid pool, fibrous cap, and calcification. They demonstrated that stent expanded asymmetrically in the axial direction because of plaque eccentricity and stiffness of fibrous capsule and calcification zones. He showed that the minimal stent length was located at the stenosed side of the plaque while the maximum length at the opposite side, and the highest the complexed plaque, the greatest the stent foreshortening [25].

Another important factor predisposing to stent elongation rather than high-pressure post-dilatation, direction of post-dilatation, malapposition of stent edge, and less plaque-media area or stent-plaque-artery complex is the number of connectors between stent hoops as it affects longitudinal stent strength. Designs with 2 connectors such as Promus and Endeavor stents are more likely to elongate [4, 10, 16] than those with more connectors such as Xience stent (3 connectors) and Cypher stent (6 connectors) [4, 16, 23].

Stent elongation and shortening in this study preserve stent geometry; however, other forms of elongation and shortening distorting stent geometry may occur and are known as LSD. The incidence of LSD is low ranging from 0.1% [5] to 1.1% [9, 10]. It can cause acute stent thrombosis, numerically high target lesion failure on long-term outcomes [14], or even death [7]. Although it may influence any stent, Promus stent is more frequently encountered in clinical studies [8, 9, 11–13]. The most common sites are ostial and bifurcation lesions especially in the left main artery [10] and are caused mainly by mechanical insults such as catheter engagement and advancing and/or withdrawing of imaging catheters (IVUS, OCT), balloon, second stent, embolic protection devices, or guidewire. The most frequent pattern is strut wrinkling, overlapping, stretching, concertina, or excessive shortening [6–8, 10]. It can be diagnosed by angiography, IVUS, OCT, and multislice computed tomography [26] and should be treated by further balloon dilatation and/or stenting.

IVUS is an important tool for the selection of stent length and assessment after stent deployment [15, 17, 27]. Grayscale IVUS gives similar results to ECG-gated IVUS on measuring stent length [28]. More recently, OCT is used as an imaging modality before and after stenting. It can be used effectively to measure stent length [17] though stent length may be shorter in some studies [29, 30]. Other modalities such as multislice computed tomography [26, 31, 32] and 3D quantitative coronary angiography (QCA) have also a good correlation with IVUS measurements [33].

## Study limitations

The study has some limitations. It is not a prospective study. The study included a real-life daily work in the catheterization room, and it is not powered to compare between the different stent types. Some manufacturers mention foreshortening in a range of percentage only and not in length (mm). The predicted calculated foreshortened length (mm) was calculated by measuring the maximum percentage. Stents longer than 20mm account for only 32% to avoid incorrect measurements. The number of stents is not large enough to calculate multivariate analysis of the difference of stent length for each stent diameter. Insurance grant affects the selection of stent type.

## Conclusions

After deployment of coronary stents in real-life coronary lesions, most of the stents are longer than the manufacturer length. It is useful to assure full lesion coverage but avoid excessive stent elongation that may protrude into the main vessel such as the left main coronary artery. This elongation is related to dilatation pressure and plaque-media at the whole lesion segment and worth mentioning in manufacturer documents.

## Data Availability

The manuscript data is available on request to the corresponding author.

## Abbreviations

A-A length: Area-to-area stent length
BMS: Bare-metal stent
DES: Drug-eluting stent
ECG: Electrocardiogram
E-E length: Edge-to-edge stent length
EEM = VA: External elastic membrane = vessel area
IQR: Interquartile range
IVUS: Intravascular ultrasound study
LA: Lumen area
LSD: Longitudinal stent deformation
NIRS-IVUS: Near-infrared spectroscopy and IVUS
OCT: Optical coherence tomography
QCA: Quantitative coronary angiography

## Declarations

The submitted article is an original article and has not been published before. The study was presented in part in ESC 2014, Barcelona, Spain (P 4520, from 30/08/2014 to 3/09/2014).
